# Ethics for check-ups

**DOI:** 10.1590/S1516-31802002000500001

**Published:** 2002-09-02

A very positive phenomenon is taking hold within the Brazilian medical scene: the idea that it is possible to get advance warning of diseases by detecting markers (biochemical, hormonal, immunological and anatomical) that would make it possible to alter the natural history of a disease. Such actions, called check-ups, screening or early detection, would avoid the catastrophic course of cancer or cardiovascular disease, for example. It must be said that a positive result from a screening test indicates a greater probability, and not the certainty that the disease will appear. Nonetheless, a question of an ethical nature arises regarding screening, which should be a matter for legitimate concern in the use that is made of it today, both inside and outside Brazil.

Longstanding medical practice has always been, and continues to be, unidirectional. That is, an individual with some discomfort seeks out a doctor to provide comfort for this condition. This means that the doctor was put to work to solve an existing problem and not to create a new one. Screening examinations invert the direction of the doctor-patient relationship. In this, doctors, clinics and laboratories actively make agreements with companies, call citizens in through the lay press, or even approach passers-by in cities, to offer “prevention” of diseases. In this way, doctors begin to interfere in the lives of individuals who had not previously felt any need for contact with such professionals. Or in other words, problems are created when they do not exist yet. Such actions are justified when the suggested tests really avoid a serious future problem, as is the case with the screening of hypothyroidism in nurseries.

Nevertheless, how much of a scientific basis do tests proclaimed as a valid act of early detection have, and how much do they represent a mixture of sincere enthusiasm with commercial interest? To answer this question, all the weapons need to be placed on the table. First, the conflict of interest must always be declared. This is always remembered in contacts between doctors and the pharmaceutical industry and forgotten in relationships between doctors and the diagnostic equipment industry. Second, the burden of proof must fall on the party that is proposing the screening test. On the contrary, as occurred with some therapeutic proposals a few decades ago, tests are first released onto the market and only afterwards are they shown not to work as proposed or to be less effective than expected. Third, the forum for debates must be within broadly based medical entities representing various specialties together, and within public-interest entities such as patients’ associations and consumer protection associations. The positions of specialists’ societies are invariably biased. Fourth, the basic criterion for the acceptance of a screening test must in most cases be the existence of controlled clinical trials that show that, when the test is applied, there is a significant reduction in mortality or a significant gain in years of good-quality life. Conclusions from observational studies can no longer be accepted.

For us to move forward with the idea that only the screening activities that have recognizable benefit may be adopted, it becomes necessary for the various sectors of the medical and scientific community to have new assignments. Faculties of medicine must expand the teaching of scientific methodology, so as to provide students with greater capacity to evaluate medical studies published within all the spheres of knowledge. The *Conselho Federal de Medicina* (Federal Medical Council), and the Regional Councils, could broaden the debate by including the Medical Ethics Committees of each hospital.

The *Associação Paulista de Medicina* (São Paulo Medical Association), through its Scientific Board, is drawing up a program of various activities to discuss the topic over the coming years, in which the specialist societies will be brought together. The journals “Diagnóstico & Tratamento” and “São Paulo Medical Journal” will always dedicate one or more articles to the theme of screening for diseases.

Lastly, it will be the responsibility of the Ministry of Health and state and municipal health departments to end their compulsive demagogy of looking for new patients through their “campaigns”, when they are not managing to solve the problems of those who have been sick for a long time. They could, in conjunction with the medical entities, create regulatory bodies for screening activities along the successful lines of the National Council for Research Ethics. Five years ago it would have been impossible to imagine that it could get Brazilian clinical research into order. Fortunately, the country is now at a civilized level in terms of clinical investigation. If the country advances in the social control of screening on an ethical and scientific basis, it will be ahead of several countries such as the United States, the European Union and Japan, where this question is avoided because of the enormous conflicts of interest that exist. After all, those countries produce more than ninety percent of all diagnostic equipment that sells the possibility of a world without illness. It is up to us to regulate the use of screening tests within our epidemiological realities and the scientific criteria. After that, we will need to avoid confusing check-ups with preventive medicine. But that is another story.

